# Spinal epidural hematoma without significant trauma in children: two case reports and review of the literature

**DOI:** 10.1186/s12887-020-1957-x

**Published:** 2020-02-19

**Authors:** Audrey Carlhan-Ledermann, Bernard Laubscher, Maja Steinlin, Christian T. Ulrich, Rajeev Kumar Verma, Mattia Rizzi, Rodolfo Maduri, Sebastian Grunt

**Affiliations:** 1 Department of Pediatrics, Hospital of Neuchâtel, Neuchâtel, Switzerland; 2 Department Woman-Mother-Child, Division of Pediatrics, University Hospital of Lausanne, Lausanne, Switzerland; 3 Division of Neuropediatrics, Development and Rehabilitation, University Children’s Hospital, Bern University Hospital, Inselspital, CH 3010 Bern, Switzerland; 4 Department of Neurosurgery, University of Bern, Inselspital, Bern, Switzerland; 5 Institute of Diagnostic and Interventional Neuroradiology, University of Bern, Inselspital, Bern, Switzerland; 6 Department Woman-Mother-Child, Division of Pediatrics, Oncology/Hematology Unit, University Hospital of Lausanne, Lausanne, Switzerland; 7 Department of Neurological Sciences, Service of Neurosurgery, University Hospital of Lausanne, Lausanne, Switzerland

**Keywords:** Spinal epidural hematoma; Hemorrhage; MRI; Paraplegia; Hemophilia; Vascular malformation

## Abstract

**Background:**

Spinal epidural hematoma without significant trauma is a rare condition with potentially severe outcome. This case report and systematic review of the literature illustrates the clinical presentation, risk factors, evaluation, treatment and outcomes of spinal epidural hematoma without significant trauma in children.

**Case presentation:**

We report one case of a 7-year-old girl who developed a neck pain after minor cervical sprain. MRI showed a right posterior epidural hematoma extending from C2/3 to T1. The hematoma was surgically evacuated, and the histopathology showed an arteriovenous malformation. Postoperative MRI showed complete evacuation of the hematoma and no residual vascular malformation. We report a second ASE with idiopathic spinal epidural hematoma of a 4½-year-old boy presenting with neck pain. MRI showed a right-sided latero-posterior subacute spinal epidural hematoma at C3–C5. Owing to the absence of any neurological deficit, the patient was treated conservatively. MRI at 3 months showed complete resolution of the hematoma.

**Conclusions:**

Spinal epidural hematoma without significant trauma in children is a rare condition. It may present with unspecific symptoms. Screening for bleeding diathesis is warranted and neuroradiologic follow-up is essential to rule out vascular malformation. Whereas most children have a favorable outcome, some do not recover, and neurological follow-up is required.

## Background

Spinal epidural hematoma (SEH) without significant trauma is rare in children. In the literature, the term “spontaneous spinal epidural hematoma” was used to describe SEH without clear traumatic etiology. Since this term, however, covers cases of idiopathic SEH, bleeding due to coagulopathy or vascular malformations and hemorrhages after minor trauma, it was recommended to avoid the terminology [1]. In the present article we therefore use the term “SEH without significant trauma”. The condition is mostly observed in adults with a bimodal distribution with peak prevalence in the 2nd and 6th decades of life with an estimated incidence of 0.1 per 100,000 patients per year [1–4]. A recent review revealed that spinal epidural hematomas occur in all age categories and are by far the most common type of spinal haematoma [3]. Most cases manifest with acute onset pain at the level of the hematoma and sensorimotor deficit with or without bladder and/or intestinal disturbances [3]. Various etiologies, such as coagulation disorders and vascular malformations have been described [3–9].

The clinical presentation of SEH without significant trauma in children is often nonspecific, including irritability, pain, torticollis, and neurological deficit [10–15]. In some cases, minimal cervical trauma precedes SEH [16, 17]. The nonspecific presentation in children may lead to delayed diagnosis and treatment. Early surgical decompression likely leads to good outcomes [18, 19]. Although surgery seems to be preferred to conservative treatment in adults, some factors are important in determining the choice of treatment: preoperative neurological status, coagulopathy and the length of SEH contribute to poor postoperative functional recovery. Literature on SEH without significant trauma in children is scarce [6, 9, 11–13, 20–30]. We report on two pediatric cases.

## Case presentation

### Case 1

**Fig. 1:**
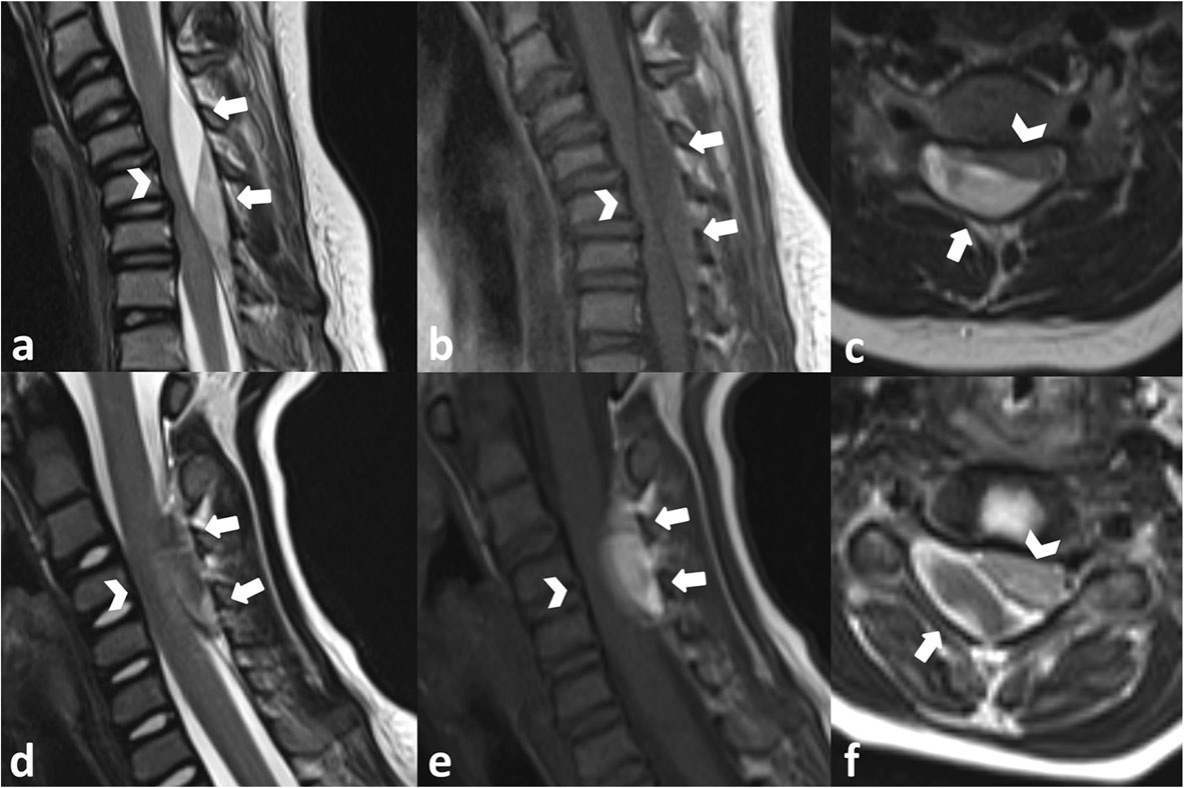
MRI scans of case 1 and case 2. In case 1 (**a**–**c**) a hyperacute epidural hemorrhage was revealed (T1w isointense signal (1b) and T2w slightly hyperintense signal (1a)). In case 2 (**d**–**f**) a subacute hemorrhage was found (T1w hyperintense (2b), and T2w iso- to slightly hyperintense signal (2a)). Arrows: epidural hemorrhage, arrowheads: spinal cord.

A 7-year-old girl without other health problems presented to the pediatric emergency department with a 10-h history of neck pain following a benign neck flexion-extension movement associated with a cracking noise. The past medical history was unremarkable. There was no a priori history of non-accidental trauma. Clinical examination revealed contractions of the para-spinal cervical muscles with no neurological deficit. Despite oral analgesia, the pain gradually increased. At the child’s second visit, no new clinical abnormalities were noted. Twenty-four hours after presentation, she developed progressive weakness of both arms and legs and paresthesia of the right hand. Bladder or sphincter dysfunction was not reported. Magnetic resonance imaging (MRI) of the spine showed hyper acute right posterior epidural hematoma extending from C2/3 to T1, leading to spinal cord compression (see Fig. 1a-c). The extensive hematologic work-up demonstrated no coagulation abnormalities. She underwent emergency right-sided C4–6 hemi-laminectomy with evacuation of the underlying hematoma. Conventional spinal and cerebral angiography was performed 2 days after evacuation of the hematoma and showed no evidence of a vascular malformation. After evacuation of the hematoma, the patient’s neurological status gradually improved, and no sensorimotor deficit was present 3 weeks postoperatively. Histopathology of the site of operation showed irregularly shaped vessel collections of venous and arterial type consistent with an arteriovenous malformation. Postoperative MRI showed complete evacuation of the hematoma and no residual vascular malformation. At 2-year follow-up, the child was neurologically intact with no clinical signs of spinal instability.

### Case 2

A 4½-year-old boy without other health problems was referred to the pediatric emergency department with a 5-day history of progressive neck pain. No history of prior trauma was reported (also not for a non-accidental trauma), and the past medical history was unremarkable. He was discharged home with oral analgesia. Persistent pain, neck stiffness, and torticollis lead to a second visit 5 days later. Physical examination on admission showed an alert child with marked torticollis, pain-induced limitation of neck movements and tenderness on palpation of the neck muscles. Neurological examination was otherwise normal. Emergency computed tomography (CT) and MRI of the spine revealed a right-sided latero-posterior subacute spinal epidural hematoma at C3–C5, exerting a mass effect on the spinal cord (see Fig. 1d-f). No vascular malformation was visible. MRI of the brain was normal. Conventional angiography was not performed. A coagulation profile revealed decreased Von Willebrand factor (VWF) levels with an antigen level of 42% and activity of 29% (normal ranges are above 50%), with an otherwise normal factor VIII level at 85% and a platelet count of 307 G/L. Owing to the absence of any neurological deficit, the patient was treated conservatively with good pain control achieved with paracetamol and morphine. Morphine was stopped after 1 week with no pain recurrence. Neck stiffness improved spontaneously. MRI at 1 week showed a decrease in the size of the hematoma. VWF level improved spontaneously, with no need for substitution. Three weeks after presentation, the child was completely asymptomatic. MRI at 3 months showed complete resolution of the hematoma. The VWF values remained within the normal range (antigen at 74% and activity at 59%). The suspicion of von Willebrand disease could be ruled out.

## Discussion and conclusion

SEH without significant trauma in children is rare. Literature on this condition is scarce and restricted to case reports, case series and narrative reviews. In order to describe clinical manifestation, risk factors and etiology, treatment options, and outcomes after SEH without significant trauma in children we present two illustrative cases. In addition, we performed a systematic literature review using PubMed (from 1950), Cochrane Library (from 1994), National Institute for Health and Care Excellence Evidence (NICE evidence: from 1999), and the Excerpta Medica Database (Embase: from 1947). The date of the last search was 24 February 2017. The following search terms were used: “spontaneous spinal epidural hematoma”, “spinal epidural hematoma”, “spontaneous spinal epidural bleeding”, and “spinal epidural bleeding”. Additional publications were identified from reference lists of selected articles. We included original studies, case reports, and case series written in English, German, French, and Spanish. Congress abstracts were not included. Only articles reporting on patients aged younger than 18 years diagnosed with SEH without significant trauma were included. Articles reporting cases of traumatic spinal epidural hematoma were excluded. Extracted data included age, symptoms and clinical findings at presentation, sex, neuroimaging studies, location of SSEH, type and timing of treatment, risk factors and etiology, and neurological findings at clinical follow-up. Furthermore, the neurological findings described at presentation and follow-up were graded using the American Spinal Injury Association (ASIA) Impairment Scale according to the International Standards for Neurological Classification of Spinal Cord Injury [31], which is a 5-level scale for assessing motor impairment in individuals with spinal cord injury.

**Fig. 2:**
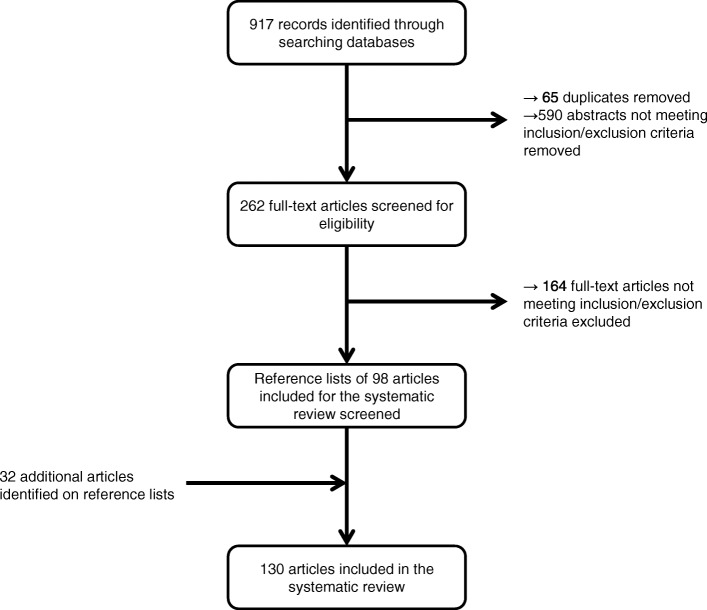
Process for selecting articles for the literature review

We identified 153 cases of children with SSEH. The search results, including the stepwise elimination process, are illustrated in Fig. 2. The details of these case reports are available in tabular form as (Additional file 1).

**Fig. 3:**
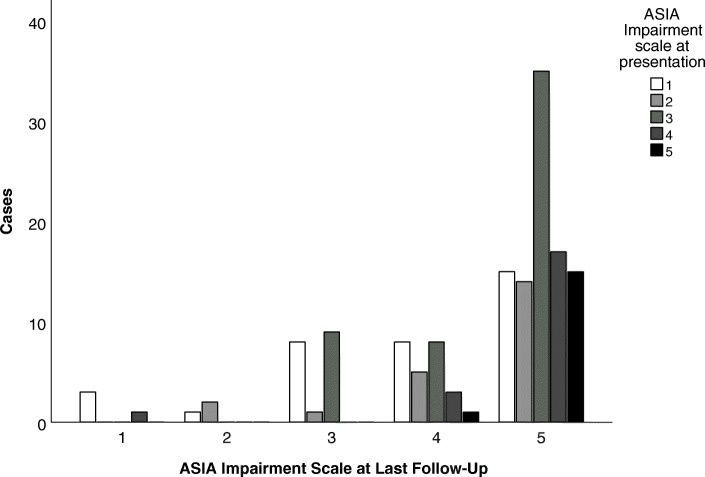
American Spinal Injury Association (ASIA) Impairment Scale at diagnosis and at last follow-up. Bar graph demonstrating the relationship of the ASIA Impairment Scale at diagnosis and at follow-up of children with SSEH

Of the 153 cases included in the final selection, 99 were boys (99/149, 66.5%) and 50 were girls (50/149, 33.5%) with a median age of 7 years (IQR [interquartile range] 20–156 months). The male to female ratio was 6.5:3.5. For 4/153 cases (2.6%), sex was not reported. Table 1 summarizes clinical signs and symptoms at presentation (time point when symptoms occurred) and at diagnosis (time point when diagnosis was made), localization, etiology, contributing factors and treatment. The retrospective ASIA impairment score at diagnosis and at last follow-up is shown in Table 2 and in Fig. 3.

**Table 1:** Patient Characteristics at Presentation

Age at diagnosis	Median 7 years (IQR 20–156 months), (*N* = 153)
Time delay (presentation to treatment)	Median 5.6 days (IQR 2.6–14.8 days), (*N* = 117)
Time delay (diagnosis to treatment)	Median 1.9 (IQR 0.7–5.7 days), (N = 117)
Symptom and signs at diagnosis	153
Pain	137 (90%)
Back pain	67 (43%)
Limb pain	15 (10%)
Abdominal pain	9 (6%)
Torticollis	67 (43%)
Irritability	38 (25%)
Tetraparesis	48 (31%)
Hemiparesis	96 (63%)
Sensory disturbances	49 (32%)
Sphincter disturbance	44 (29%)
Neurological deficit at initial presentation	104 (68%)
Neurological deficit at diagnosis	136 (89%)
Neuroimaging studies	144
MRI	83 (58%)
CT	15 (10%)
Myelography	30 (21%)
MRI and CT	14 (10%)
CT and myelography	2 (1%)
Localization	149
Cervical	21 (14%)
Cervico-thoracic	82 (55%)
Thoracic	37 (25%)
Thoraco-lumbar	4 (2.5%)
Lumbar	4 (2.5%)
Lumbo-sacral	1 (1%)
Antero-posterior localization	132
Posterior	112 (85%)
Anterior	17 (13%)
Lateral	3 (2%)
Etiology and contributing factors	150
Unknown	57 (38%)
Trivial trauma	30 (20%)
Vascular malformation	21 (14%)
Arteriovenous malformation	10 (48%)
Venous angioma	4 (19%)
Angiolipoma	2 (9%)
Hemangioma	4 (19%)
Arteriovenous fistula	1 (5%)
Coagulation disorder	42 (28%)
Hemophilia A	27 (64%)
Hemophilia B	12 (29%)
Hemophilia A and B	1 (2%)
Coagulopathy due to cholestasis	1 (2%)
Unknown hemophilia	1 (2%)
Treatment	151
Surgery and factor replacement	12 (8%)
Factor replacement without surgery	36 (24%)
Surgery without factor replacement	103 (68%)

*CT* computed tomography, *IQR* interquartile range, *MRI* magnetic resonance imaging

**Table 2:** American Spinal Injury Association (ASIA) Impairment Scale (AIS) Score at Diagnosis and at Last Follow Up

Score		Explanation	At Diagnosis	At Last Follow-Up
A	Complete	No motor or sensory function is preserved in the sacral segments S4–S5	38/153 (25%)	3/148 (2%)
B	Incomplete	Sensory but no motor function is preserved below the neurological level and includes the sacral segments S4–S5	26/153 (17%)	8/148 (5.5%)
C	Incomplete	Motor function is preserved below the neurological level, and more than half of key muscles below the neurological level have a muscle grade less than 3	51/153 (33.5%)	14/148 (9.5%)
D	Incomplete	Motor function is preserved below the neurological level, and at least half of key muscles below the neurological level have a muscle grade of 3 or more	19/153 (12.5%)	27/148 (18%)
E	Normal	Motor and sensory function are normal	17/153 (11%)	96/148 (65.5%)

SEH without significant trauma in children may manifest with local pain before any progressive neurological deficit due to spinal cord compression is evident. The classical triad is severe localized spinal pain, radicular pain, and sensorimotor deficit. In contrast to adults [4], manifestation of SEH without significant trauma in children is less specific. The present review confirmed that children below 2 years present mainly with irritability (84%). The diagnosis of SEH without significant trauma in children, especially in toddlers, is challenging. Appropriate treatment may be delayed. This can potentially lead to long-term residual neurological deficits. We found a median delay between presentation and diagnosis was 3.7 days, and median delay between presentation and treatment of 5.6 days. We consider that in children presenting with unspecific symptoms, such as irritability and torticollis, clinical awareness and urgent neuroradiologic evaluation with full spine MRI is essential to rule out SEH. Whereas, in the older publications, myelography and CT was the imaging method of choice, a considerable increase of MRI-related diagnosis of SEH without significant trauma over the past 15 years was evident. The review findings suggest that with increasing availability of imaging facilities (CT, MRI), the number of publications on SSEH cases has increased and patients seem to be treated earlier. However, the no conclusion can be drawn regarding time-trends of treatments and prognosis of the condition.

Trivial spine trauma (minimal effort, sports, neck sprain, fall from own height) preceded the SEH in 20% of all cases. In 55% of the children with cervical SEH, minor spinal trauma preceded the bleeding. Vascular malformation (diagnosed by MR-angiography, during operative evacuation of the hematoma or from histopathology) was found as the cause of SEH in 14%. Arteriovenous malformation was the most frequently reported type of vascular malformation. MRI is considered the gold standard for the diagnosis of SEH and the first choice in the diagnostic work-up. As MRI may not always rule out vascular malformations, a conventional spinal angiogram is essential in negative cases. The use of spinal angiograms was only reported in a few instances. Vascular malformations may be underdiagnosed in children with SEH without significant trauma, especially in cases with preceding minor trauma. In our first case, there was no evidence of vascular malformation in MRI and conventional angiography, but histopathological signs of an arteriovenous malformation were found in evacuated material. We assume that the vascular malformation was compacted due to the space occupying effect of the hematoma. Hence, the rupture of a pre-existing arteriovenous malformation triggered by minor trauma is the most likely diagnosis in case 1. Like Sivakumaran, King, Bodi, Chandler and Walsh [32] we recommend performing a conventional spinal angiogram in all patients who have SEH without significant trauma but no evidence of a coagulation disorder. A coagulation disorder was found in 28% of the cases reviewed and hemophilia A was the most frequent abnormality. Although the severity of hemophilia was mostly not specified, we assume that most patients with SEH without significant trauma were diagnosed with mild forms and SEH was the first manifestation of the coagulation disorder. Children with coagulation disorders diagnosed with SEH without significant trauma were younger than those who had SEH without coagulation disorder (54 months [IQR 10–84] vs 96 months [IQR 24–168]). None of the children was reported to be under treatment with platelet aggregation inhibitors or anticoagulants. Whether a combination of bleeding diathesis and vascular pathologies increases the risk for SEH in children remains unknown. Nevertheless, coagulation studies and a thorough neuroradiologic work-up are essential in all children with SEH without significant trauma.

Before surgical decompression, factor replacement is the most important treatment for SEH due to coagulation disorders, depending on the severity of the disorder, to guarantee adequate hemostasis and to prevent hematoma progression and further neurological aggravation. We found that median treatment delay was shorter in patients with coagulation disorder (4 days compared to 10 days). We assume that the clinicians’ alertness to SEH in patients with bleeding diathesis is greater, leading to a prompter diagnosis. In general, SEH represents a surgical emergency. However, case 2 illustrates that not all cases with SEH without significant trauma in children require a surgical approach. In the literature review 36 of 151 patients did not undergo surgery, because of factor replacement for hemophilia (27 cases), rapid resolution of symptoms (4 cases), or no neurological deficit (5 cases). In cases (such as in our case 2) with long hematoma (less compressive) and in stable patients with minimal neurological deficit, or in cases with early resolution of the hematoma, conservative management may be considered [3, 14, 15, 20, 33]. The exact mechanism of blood clot dissolution in case 2 remains unclear. Various mechanisms - such as redistribution of bleeding, or absorption of the clot by proliferation of endothelial cells or dural supportive tissue - may be responsible.

Two percent of the patients with SEH described in the literature died. We found that 65.5% of the surviving patients of children (aged under 18 years) with SEH without significant trauma recovered completely (see Table 2) – in contrast to the lower overall complete resolution rate of 40% previously reported for all age categories (0–90 years) [4]. However, spinal cord compression within the context of SEH may lead to residual neurological deficits with considerable life-long morbidity in children, and even to death. In particular, children with more severe impairment at presentation seem to be more likely to have more severe long-term impairment, including bladder or sphincter dysfunction. Figure 3 shows the relationship between the ASIA impairment scale scores at last follow-up and at diagnosis (see Fig. 3). Long-term neurological follow-up, including bladder-function monitoring and neuroimaging is necessary, even for children who are asymptomatic at hospital discharge.

To conclude, literature regarding SEH without significant trauma in children is scarce and restricted to case reports and case series. There is a lack of higher-level evidence. Children with SEH without significant trauma often present with unspecific symptoms, such as irritability. This may delay diagnosis and timely initiation of treatment, especially in young children and infants. After a trivial cervical trauma and increasing local pain, clinicians can be falsely reassured and diagnose a trivial torticollis, like in our case. Any progressive cervico-thoracic pain, even after trivial traumas, should alert clinicians. The most common contributory factors are bleeding diathesis and vascular malformation. Therefore, coagulation studies are essential in every child with SEH without significant trauma. MRI is the neuroradiologic procedure of choice in these children. However, as MRI may not rule out vascular malformation, conventional angiography should be considered for this purpose.

## Supplementary information

**Additional file 1.** Alphabetical list of authors of articles included in the review and summary of study design and findings.

## Data Availability

All data generated or analysed during this study are included in this published article.

## Abbreviations

ASIA: American Spinal Injury Association
CT: Computed tomography
IQR: Interquartile range
MRI: Magnetic resonance imaging
SEH: Spinal epidural hematoma
VWF: Von Willebrand factor
