# Supplementary material for: Spinal epidural hematoma without significant trauma in children: two case reports and review of the literature
Source: BMC Pediatr. 2020 Feb 19;20:77. doi: 10.1186/s12887-020-1957-x (PMC7029477; doi:10.1186/s12887-020-1957-x)
Supplement: Supplementary file 1 — Additional file 1. Alphabetical list of authors of articles included in the review and summary of study design and findings. [file 12887_2020_1957_MOESM1_ESM.pdf]

Appendix 1. Alphabetical list of Authors of Articles Included in the Review and Summary of Study Design and Findings

Study	Level of Evidence	Research Design	Number of Cases/Number of Episodes; Case Series: Number Included	Sex	Age (Months) Case Series: Median Age/Age Range	Symptoms at Diagnosis	Location	Risk Factors	Treatment	Outcome
Abbas et al[1]	V	Case report	1	M	5	Irritability, torticollis, upper limb paraplegia, lower limb paraparesis	C4-C7, anterior	Trivial trauma (fall from 40 cm)	Laminectomy	NA
Abdelaal et al[2]	V	Case report	1	M	60	Torticollis, lower limb hyperreflexia	C1-T8, posterior	Hemophilia A	Factor VIII + laminectomy	No sequelae
Abram et al[3]	V	Case report	1/3	F	120	100% paraplegia; 33% back pain, anesthesia	1. NA<br>2. NA<br>3. C7-T1, posterior	33% none, 66% NA	66% conservatively; 33% laminectomy	Paraplegia and anesthesia below T5
Agha et al[4]	V	Case report	1	M	10	Irritability, torticollis	C1-L4, posterior	Hemophilia A	Factor VIII	No sequelae
Amyot et al[5]	V	Case report	1	NA	22	Torticollis, Erb palsy	C2-T1, posterior	Trivial trauma (fall from his own height)	Laminectomy	Left upper limb paresis
Arya et al[6]	V	Case report	1	F	12	Irritability, lower limb paraplegia, upper limb paraparesis	C2-C6, posterior	None	Conservatively	Tetraparesis
Awada et al[7]	V	Case report	1	M	168	Torticollis, tetraplegia, anesthesia below C5, sphincter dysfunction	C2-T2, posterior	None	Laminectomy	Tetraplegia, anesthesia below C7
Azumagawa et al[8]	V	Case report	1	M	144	Torticollis, back pain, limb pain, left hand paresis	C4-T4, posterior	None	Conservatively	No sequelae

Balkan et al[9]	V	Case report	1	M	204	Torticollis, back pain, tetraparesis, lower limb anesthesia, sphincter dysfunction	C6/7-T5 anterior, T5-T12 posterior	Hemophilia B	Factor IX	No sequelae
Bisson et al[10]	V	Case report	1	M	84	Torticollis, limb pain	C2-T3, anterior	Hemophilia B	Factor IX	No sequelae
Blount et al[11]	V	Case report	1	M	0	Tetraplegia at birth	C5-T6, posterior	None	Laminotomy	Tetraplegia
Borkar et al[12]	V	Case report	1	M	60	Back pain, paraparesis, hypoesthesia below L1, sphincter dysfunction	L4-S2, anterior	Hemophilia B	Factor IX	No sequelae
Cabral et al[13]	V	Case report	1	M	108	Torticollis, back pain, paraplegia	C7-T4, posterior	Arteriovenous malformation	Laminectomy	No sequelae
Cakir et al[14]	V	Case report	1	F	108	Limb pain, hypoesthesia under T3, paraplegia	T2-T8, posterior	Trivial trauma	Laminectomy	No sequelae
Caldarelli et al[15]	IV	Case series	2; 2	50% M, F	18.5/16–24	100% hemiparesis; 50% irritability, torticollis, abdominal pain	1. C4-C7, posterior<br>2. C5-T4, latero-posterior	100% none	100% laminotomy	100% no sequelae
Carrea et al[16]	IV	Case series	3; 1	F	48	Back pain, paraparesis, sphincter dysfunction	T5-T10	Trivial trauma (fall from his own height)	Laminectomy	No sequelae
Chang et al[17]	V	Case report	1	F	60	Abdominal pain, torticollis, paraparesis, sphincter dysfunction	T6-T8, postero-lateral	None	Laminectomy	No sequelae
Chrétiennot-Bara et al[18]	IV	Case series	2; 2	100% M	54/12–96	100% paresis; 50% abdominal pain, back pain, irritability, hypoesthesia	1. C7-L5, posterior<br>2. T11-L5	100% coagulopathy (50% hemophilia B, hemophilia A)	50% factor; 50% factor + laminectomy	100% no sequelae

Cirek et al[19]	V	Case report	1	M	96	Torticollis, tetraplegia	C3-C7, posterior	None	Laminectomy	No sequelae
Cooper[20]	V	Case report	1	F	168	Torticollis, tetraplegia, anesthesia below T5	C3-C5	None	Laminectomy	No sequelae
Cromwell et al[21]	V	Case report	1	M	18	Irritability, torticollis, tetraparesis	C2-T8	Hemophilia A + trivial trauma	Factor VIII, laminectomy	Tetraparesis
Cuvelier et al[22]	V	Case report	1	M	10	Irritability, torticollis, upper paraplegia, lower paraparesis	C2-T8, posterior	Hemophilia A	Factor VIII, laminectomy	No sequelae
Dauch[23]	V	Case report	1	M	132	Intermittent back pain, paraparesis	T1-T3, posterior	None	Laminectomy	Paraparesis
Dawson[24]	IV	Case series	2; 2	50% M, F	150 / 120-180	100% back pain; 50% tetraplegia, hypoesthesia	1. T2-T6<br>2. L2-L5	50% arteriovenous malformation, arteriovenous malformation + trivial trauma	100% laminectomy	100% no sequelae
Doymaz et al[25]	V	Case report	1	M	168	Back pain, tetraplegia, anesthesia	C1-T2, anterior	Coagulopathy	Laminectomy, transfusion	Tetraparesis
Erkutlu et al[26]	V	Case report	1	M	60	Irritability, torticollis, paraplegia, anesthesia in lower limbs, sphincter dysfunction	T9-T10, posterior	Hemophilia A + trivial trauma	Activated prothrombin complex concentrate	Paraparesis
Fok et al[27]	V	Case report	1	M	15	Torticollis, upper limb paraplegia, lower limb paraparesis	C3-T8, posterior	None	Laminectomy	No sequelae
Fruchtman et al[28]	V	Case report	1	M	6	Irritability, pain	T7-T10, posterior	Hemophilia A	Factor VIII	No sequelae
Gelabert et al[29]	IV	Case series	8; 1	F	48	Back pain, paraparesis	T2-T5, posterior	Angiolipoma	Laminectomy	No sequelae
Ghanem et al[30]	V	Case report	1	M	96	Back pain, paraplegia, anesthesia,	T1-T3, posterior	Trivial trauma (lifting weight)	Laminectomy	No sequelae

						sphincter dysfunction				
Grollmus[31]	IV	Case series	2; 1	NA	180	Back pain, limb pain, tetraplegia	C6-T1, posterior	None	Laminectomy	No sequelae
Gupta et al[32]	V	Case report	1	M	72	Torticollis, tetraparesis	C3-T4	Trivial trauma (blow on the neck)	Laminectomy	No sequelae
Güzel et al[33]	V	Case report	1	F	168	Back pain, upper paraparesis, lower paraplegia, anesthesia below T6, sphincter dysfunction	C4-T6, posterior	Trivial trauma (convulsion)	Laminectomy	Paraparesis, sphincter dysfunction
Hamre et al[34]	V	Case report	1	M	108	Torticollis, back pain, paresis, hypoesthesia, sphincter dysfunction	C2/3-T6 anterior	Hemophilia B	Factor IX	No sequelae
Hasni Bouraoui[35]	IV	Case series	3; 1	M	156	Torticollis, back pain, paraparesis	T2-T6, posterior	None	Laminectomy	No sequelae
Heer et al[36]	V	Case report	1	M	144	Torticollis, tetraparesis, sphincter dysfunction, anesthesia below T4	C5-C6, posterior	Hemophilia A (unknown)	Laminectomy, factor VIII	Tetraparesis, sphincter dysfunction
Hehman[37]	V	Case report	1	M	16	Irritability, torticollis, tetraparesis	C6-T9, posterior	Trivial trauma	Laminectomy	No sequelae
Horcajadas et al[38]	IV	Case series	2; 1	F	48	Back pain, limb pain, torticollis, paraplegia, hypoesthesia, sphincter dysfunction	T2-T4, posterior	None	Laminectomy	No sequelae
Hosoki et al[39]	V	Case report	1	M	11	Irritability, torticollis, tetraparesis	T9-L1, posterior	None	Laminectomy	No sequelae
Hutt et al[40]	V	Case report	1	M	11	Irritability, abdominal pain	T9-T12, posterior	Hemophilia A	Factor VIII	No sequelae

Iguchi et al[41]	V	Case report	1	F	14	Paraplegia	T3-T6, posterior	None	Laminectomy	No sequelae
Iizuka et al[42]	V	Case report	1	M	60	Torticollis, back pain, tetraplegia	C2-T1, anterior	Hemophilia A	Factor VIII, laminoplasty	No sequelae
Irwin et al[43]	V	Case report	1	M	10	Irritability, torticollis, back pain, abdominal pain, paraparesis, sphincter dysfunction	C4-L1, posterior	Hemophilia A	Factor VIII, laminectomy	No sequelae
Iwamuro et al[44]	V	Case report	1	M	7	Irritability, abdominal pain, paresis	T1-L1, posterior	Hemophilia B	Factor IX	No sequelae
Jackson[45]	V	Case report	1	F	168	Paraplegia	C1-C7, anterior	None	Autopsy	Death
Jackson[46]	V	Case report	1	F	168	Paraparesis, lethargy	T1-T5, posterior	None	Laminectomy	No sequelae
Johnston[47]	V	Case report	1	M	60	Abdominal pain, paraplegia	T9-T11 posterior	Hemangioma	Autopsy	Death
Jones et al[48]	IV	Case series	3; 1	M	144	Torticollis, back pain, paraplegia	C6-T1, posterior	Hemophilia	Laminectomy	Paraplegia
Joseph et al[49]	V	Case report	1	F	204	Back pain, paraplegia, hypoesthesia below T4, sphincter dysfunction	C7-T2, posterior	None	Laminectomy	Paraparesis
Jost et al[50]	V	Case report	1	M	84	Thoracic back pain	T10-T11, posterior	Hemophilia B	Factor IX	No sequelae
Jumani et al[51]	V	Case report	1	M	24	Torticollis, right hemiparesis	C4-T7, posterior	None	Laminectomy	No sequelae
Kalina et al[52]	V	Case report	1	M	7	Irritability, torticollis, paraparesis	C2-L4, posterior	Hemophilia A (unknown)	Factor VIII	No sequelae
Khursheed et al[53]	V	Case report	1	M	48	Back pain, paraparesis, hypoesthesia below T7, sphincter dysfunction	T6-T8, posterior	Hemophilia A	Factor VIII, Laminotomy	Paraparesis

Khursheed et al[54]	IV	Case series	2; 2	100% M	174 / 168-180	100% hemiparesis, anesthesia, sphincter dysfunction; 50% back pain, torticollis	1. T6-T8, posterior 2. C6-C7, posterior	100% trivial trauma	100% laminectomy	50% no sequelae, paraparesis, sphincter dysfunction
Kiehna et al[55]	V	Case report	1	M	5	Irritability, torticollis, tetraparesis	C1-conus, posterior	Hemophilia A	Factor VIII	No sequelae
Kirwan et al[56]	V	Case report	1	M	120	Torticollis, limb paresis	C5-T1, lateral	Arteriovenous malformation	Laminectomy	No sequelae
Kitagawa et al[57]	V	Case report	1	F	144	Back pain, paraplegia, anesthesia below T4, sphincter dysfunction	T1-T3, latero-posterior	Arteriovenous malformation	Laminectomy	Paraparesis
Kotilainen et al[58]	V	Case report	1	M	156	Pain, paresthesia	L4-L5, lateral	Trivial trauma	Laminectomy	No sequelae
Kubota et al[59]	V	Case report	1	M	4	Irritability, hemiplegia	C4-S1, posterior	Hemophilia A (unknown)	Factor VIII	No sequelae
Lannum et al[60]	V	Case report	1	M	204	Back pain, paraplegia, anesthesia below T6	T2-T3, posterior	Arterio-venous fistula	Laminectomy	Paraparesis
Lee et al[61]	V	Case report	1	M	4	Irritability, torticollis, tetraplegia	C4-T4, latero-posterior	None	Laminectomy	No sequelae
Lee et al[62]	V	Case report	1	M	32	Torticollis, paraplegia	C4-T3, posterior	None	Laminectomy	No sequelae
Lehman et al[63]	V	Case report	1	M	144	Back pain, paraplegia, hypoesthesia below T4	C7-T4, posterior	Trivial trauma	Laminectomy	Paraplegia
Lepintre et al[64]	IV	Case series	3; 3	33% M; 66% F	72 / 17-96	100% tetraplegia; 33% torticollis, sphincter dysfunction	1. C3-C7, posterior 2. C2-T2 posterior 3. C7-T1 posterior	33% trivial trauma, hemophilia, none	100% laminectomy	66% no sequelae; 33% tetraplegia
Lepoire et al[65]	IV	Case series	4; 1	M	180	Back pain, paraplegia	C6-T1, posterior	None	Laminectomy	No sequelae

Liao et al[66]	IV	Case series	17; 1	M	24	Torticollis, tetraplegia	C2-T4, posterior	Trivial trauma	Laminectomy	No sequelae
Liao et al[67]	IV	Case series	35; 1	F	7	Irritability, paraplegia	C7-T7, posterior	None	Laminectomy	Paraplegia
Licata et al[68]	IV	Case series	10; 1	M	18	Tetraparesis	T1-T2	None	Laminectomy	No sequelae
Lim et al[69]	V	Case report	1	F	20	Torticollis, paraparesis, sphincter dysfunction	C7-T4, posterior	None	Laminectomy	Paraparesis
Lo[70]	IV	Case series	2	100% M	102 / 72-132	100%: paraplegia, anesthesia, sphincter dysfunction; 50% back pain	1. T3-T5, posterior 2. C7-T4 posterior	100% hemangioma	100% laminectomy	100% paraparesis; 50% sphincter dysfunction, hypoesthesia
Matsume et al[71]	V	Case report	1 / 2	M	156	100% limb pain, paraparesis, hypoesthesia, sphincter dysfunction; 50% paraplegia	1. NA 2. C4-C7 posterior	50% NA, none	50% conservatively, laminectomy	50% no sequelae, paraparesis
Mayer et al[72]	IV	Case series	4; 1	F	204	Back pain, paraplegia, Horner syndrome	C7-T1, posterior	Angioma	Laminectomy	Paraplegia
Min et al[73]	V	Case report	1	M	8	Irritability, paraplegia	C7-T3, posterior	Vascular malformation	Laminectomy	No sequelae
Miyagi et al[74]	V	Case report	1	M	192	Torticollis, tetraplegia, hypoesthesia below C4	C2-C6, posterior	Vascular malformation	Laminectomy	No sequelae
Morsing et al[75]	V	Case report	1	M	13	Irritability, torticollis, back pain, abdominal pain, paraparesis, Horner syndrome	C1-sacrum, posterior	Hemophilia A	Factor VIII	No sequelae
Muhonen et al[76]	V	Case report	1	M	22	Irritability, tetraparesis	C5-T2, posterior	Vascular malformation	Laminectomy	No sequelae
Nagel et al[77]	V	Case report	1	F	84	Torticollis, back pain,	C4-C5, posterior	Trivial trauma	Laminectomy	No sequelae

						limb pain, paresis				
Narawong et al[78]	IV	Case series	2; 2	50% M, NA	28 / 8-48	100% torticollis; 50% back pain, limb pain, irritability	1. T1-T5, posterior 2. C2-T3, posterior	50% hemophilia A+ trivial trauma, hemophilia A	100% factor VIII	100% no sequelae
Nayak et al[79]	V	Case report	1	F	144	Back pain, paraplegia, anesthesia below T4, sphincter dysfunction	C7-T3, posterior	None	Laminectomy	Paraparesis, anesthesia below T10
Nichols et al[80]	V	Case report	1	M	180	Back pain, paraplegia, anesthesia below T4	C6-T1, posterior	Vascular malformation	Laminectomy	No sequelae
Nirupam et al[81]	V	Case report	1	M	108	Abdominal pain, paraplegia, anesthesia, sphincter dysfunction	C6-T12, posterior	Hemophilia B	Factor IX, surgery	Death
Noth et al[82]	V	Case report	1	M	6	Irritability, torticollis, upper paraplegia, lower paraparesis	C2/3-T6, posterior	Hemophilia A	Factor VIII	No sequelae
Ohegbulam et al[83]	IV	Case series	3; 1	M	180	Back pain, limb pain	L3-L4, latero-posterior	Trivial trauma	Conservatively	No sequelae
Oymak et al[84]	V	Case report	1	M	10	Irritability, torticollis, paraparesis	C1-conus, posterior	Hemophilia A	Factor VIII, VIIa	No sequelae
Packer et al[85]	IV	Case series	2; 1	50% M, F	180 / 156-204	100% back pain, paraplegia, anesthesia	1. C7-T7 2. T9, posterior	100% vascular malformation	100% laminectomy	50% no sequelae, paraparesis
Pai et al[86]	V	Case report	1	M	15	Irritability, upper paraplegia, lower paraparesis	C4-T3, posterior	None	Laminotomy	No sequelae
Paraskevopoulos et al[87]	V	Case report	1	M	96	Paraplegia	C6-T2, posterior	Vascular malformation	Laminectomy	Paraparesis
Patel et al[88]	V	Case report	1	M	18	Irritability, torticollis,	C1-T2, posterior	None	Laminectomy	Tetraparesis

						upper paraplegia, lower paraparesis				
Patel et al[89]	IV	Case series	2; 2	50% M; 50% F	35; / 22- 48	50% irritability, paraparesis, torticollis	1. C7-T10, posterior 2. C7-T1, anterior	50% none, trivial trauma	50% laminectomy, conservatively	100% no sequelae
Pear[90]	IV	Case series	8; 1	M	180	Back pain, paraparesis	T5-T7	Trivial trauma	Laminectomy	No sequelae
Pecha et al[91]	V	Case report	1	M	168	Back pain, lower paraplegia, upper paraparesis	T1-T4, posterior	None	Laminectomy	Paraparesis
Per et al[92]	IV	Case series	10; 1	M	60	Torticollis, back pain	C3-T2, posterior	Hemophilia A	Factor VIII	No sequelae
Poonai et al[93]	V	Case report	1	F	11	Irritability, paraplegia	C4-T3, posterior	None	Laminectomy	Paraparesis
Posnikoff[94]	V	Case report	1	F	30	Irritability, torticollis, back pain, tetraparesis	C5-T5, posterior	None	Laminectomy	No sequelae
Rajz et al[95]	IV	Case series	17; 2	100% F	144 / 120-168	100% back pain, paraplegia; 50% hypoesthesia	1. T2-T4, posterior 2. T9-T11, posterior	100% trivial trauma	100% laminectomy	50% no sequelae, paraparesis
Ramelli et al[96]	IV	Case series	2; 2	50% M, F	84,5 / 7-162	50% irritability, back pain, paraplegia, tetraplegia	1. C5-T2, posterior 2. C6-T7, posterior	50% synovial cyst, none	100% laminectomy	50% paraparesis, paraplegia
Rao et al[97]	V	Case report	1	M	204	Paraparesis	L4-L5	Trivial trauma	Laminectomy	No sequelae
Rathi[98]	V	Case report	1	M	3	Tetraparesis, hypoesthesia, sphincter dysfunction	C2-T5, posterior	Hemophilia A (unknown)	Factor VIII	No sequelae
Ratre et al[99]	V	Case report	1	M	168	Back pain, paraplegia, anesthesia below T4, sphincter dysfunction	T1-T2, anterior	None	Laminectomy	Paraplegia, anesthesia below T4, sphincter dysfunction

Ravid et al[100]	V	Case report	1	F	120	Limb pain, paresis, hypoesthesia	C6-T1, antero-lateral	Trivial trauma	Laminectomy	No sequelae
Rebello et al[101]	V	Case report	1	M	132	Torticollis, back pain, tetraplegia	C3-C7, posterior	Trivial trauma	Laminectomy	Paraparesis
Robertson et al[102]	V	Case report	1	F	72	Back pain, paraplegia, hypoesthesia below T4	T1-T3	None	Laminectomy	Paraparesis
Rois et al[103]	V	Case report	1	M	156	Back pain, paraparesis	T5-T6, posterior	Hemophilia A + trivial trauma	Factor VIII, laminectomy	No sequelae
Rosenberg et al[104]	V	Case report	1	F	168	Back pain, paraparesis, sphincter dysfunction	T3-T5, posterior	Trivial trauma	Laminectomy	No sequelae
Sandvik et al[105]	V	Case report	1	M	12	Paraplegia	C6-T7, posterior	Cavernous angiolipoma	Laminotomy	No sequelae
Sankar et al[106]	V	Case report	1	M	48	Torticollis	C2-C7, anterior	Hemophilia B (unknown)	Factor IX	NA
Scheil et al[107]	IV	Case series	7; 1	M	192	Torticollis, back pain, tetraplegia, anesthesia below T3	C7-T2, posterior	None	Laminectomy	No sequelae
Schoonjans et al[108]	V	Case report	1	M	8	Irritability, paraplegia, sphincter dysfunction	C5-L1, posterior	None	Laminectomy	Tetraparesis
Scott[109]	V	Case report	1	F	108	Back pain, limb pain, paraplegia, anesthesia below T4, sphincter dysfunction	T1-T4, posterior	None	Laminectomy	No sequelae
Sheikh et al[110]	V	Case report	1	M	84	Back pain, hemiparesis	C3-L5, posterior	Hemophilia B, trivial trauma	Factor IX	No sequelae
Shenkin et al[111]	V	Case report	1	M	20	Irritability, paraplegia	C7-T7	None	Laminectomy	No sequelae
Shin et al[112]	IV	Case series	14; 1	M	204	Tetraplegia, anesthesia	C5-C6, posterior	None	Laminectomy	Tetraparesis
Shin et al[113]	V	Case report	1 / 3	F	192	100% torticollis, pain; 33%	C7-T2 anterior	None	66% laminectomy, 33% conservative	66% no sequelae; 33% paraparesis, sphincter dysfunction, syringomyelia

						tetraparesis, hypoesthesia below C7, sphincter dysfunction				
Sivakumaran et al[114]	IV	Case series	2; 2	50% M, F	126 / 96-156	100% sphincter dysfunction 50% torticollis, back pain, tetraparesis, paraplegia,	1. C4/5-C7/T1, posterior 2. C7/T1-T4/5	100% arteriovenous malformation	50% laminectomy, laminotomy	50% no sequelae, paraparesis
Soundappan et al[115]	V	Case report	1	F	11	Irritability, torticollis, paraplegia	C6-T7, posterior	Trivial trauma	Laminectomy	Paraparesis, Horner syndrome
Stanley et al[116]	V	Case report	1	M	156	Back pain, limb pain, paraparesis, hypoesthesia below T8	T5-T8, anterior	Hemophilia A (unknown), trivial trauma	Factor VIII, laminectomy	No sequelae
Tagaya et al[117]	V	Case report	1	M	4	Irritability, hemiparesis	C2/3-L2, posterior	Hemophilia A (unknown)	Factor VIII	No sequelae
Tamura et al[118]	V	Case report	1	M	60	Torticollis, paraparesis, sphincter dysfunction	C7-T1, latero-posterior	Hemophilia A	Factor VIII, laminectomy	Paraparesis
Tender et al[119]	V	Case report	1	F	144	Torticollis, tetraparesis, sphincter dysfunction	C6-T2, posterior	None	Laminectomy	No sequelae
Tewari et al[120]	IV	Case series	3; 3	66% M; 33% F	96 / 60-132	100% sphincter dysfunction; 66% torticollis, back pain, tetraplegia; 33% limb pain, paraparesis	1. C4-C7 2. C5-T4 3. T10-L4	66% none; 33% trivial trauma	100% laminectomy	66% tetraparesis; 33% paraparesis
Travis et al[121]	V	Case report	1	M	20	Irritability, paraparesis	C5-Cauda equina	Hemophilia A	Factor VIII, laminectomy	No sequelae
Valladares[122]	IV	Case series	2; 2	50% M, F	31,5 / 30-33	50% paraplegia, tetraplegia	1. T3-T5 2. C7-T3	50% none, trivial trauma	100% laminectomy	50% no sequelae, tetraparesis

Vallee et al[123]	V	Case report	1	F	22	Irritability, torticollis, tetraparesis	C5-T1	None	Laminectomy	No sequelae
Van Heesewijk et al[124]	V	Case report	1	F	48	Torticollis, paresis	C2-C6, posterior	None	Laminectomy	No sequelae
Ventureyra et al[125]	V	Case report	1	M	96	Back pain, paraplegia, sphincter dysfunction	T1-T3, posterior	Trivial trauma	Laminectomy	No sequelae
Visconti et al[126]	IV	Case series	8; 1	NA	84	Paraplegia, sphincter dysfunction	NA	Hemophilia A+B + trivial trauma	Factor VIII, IX,	Paraparesis
Williams et al[127]	IV	Case series	2; 1	F	84	Torticollis, tetraparesis, Horner syndrome	C4-T2, latero-posterior	None	Laminectomy	Tetraparesis
Wittebol et al[128]	IV	Case series	4; 1	F	72	Torticollis, back pain, tetraplegia, sphincter dysfunction	C6-T5, posterior	None	Laminectomy	Tetraparesis
Yildiz et al[129]	V	Case report	1	F	96	Back pain, paraplegia, sphincter dysfunction	T2-T4, latero-anterior	None	Laminectomy	No sequelae
Zhong et al[130]	V	Case report	1	M	84	Torticollis, paraparesis	C2-T1, anterior	Hemophilia B + trivial trauma	Factor IX	No sequelae
Presenting cases	III	Systematic reviews of case studies	2	50% M, F	69,5 / 55-84	100% torticollis; 50% tetraparesis, sphincter dysfunction	1. C2/3-T1, latero-posterior 2. C3-C5, lateror-posterior	50% arteriovenous malformation + trivial trauma, none	50% laminectomy, conservative	100% no sequelae

NA = Not available

Alphabetical reference list of cases

1. Abbas A, Afzal K, Mujeeb AA, et al. Spontaneous ventral spinal epidural hematoma in an infant: An unusual presentation. *Iran J Child Neurol* 2013;7:47-50.

2. Abdelaal MA, McGuiness FE, Sagar G. Case report: spinal extradural haematoma in haemophilia A – A diagnosis not to be missed. *Clin Radiol* 1994;49:573-75.

3. Abram HS, DeLaHunt MJ, Merinbaum DJ, et al. Recurrent spontaneous spinal epidural hematoma in a child: First case report. *Pediatr Neurol* 2007;36:177-80.

4. Agha BS, Tehan C, Perno J. Torticollis, not always the usual suspects. *Pediatr EmergCare* 2011;27:32-33.

5. Amyot R, Hardy J, Chagnon J, et al. Hématome epidural rachidien spontané chez un enfant de 22 mois. *L'Union médicale du Canada*. 1969;98:1302-06.

6. Arya R, Jain P, Kumar A, Gulati S. Spontaneous Spinal Epidural Hematoma in an Infant. *J Child Neurol* 2012;27:1577-79.

7. Awada A, Russel N, Al Fayez N, et al. Spontaneous cervical epidural hematoma: case report. *Spinal Cord* 1998;36:71-72.

8. Azumagawa K, Yamamoto S, Tanaka K, et al. Non-operative treated spontaneous spinal epidural hematoma in a 12-year-old boy. *Pediatr Emerg Care* 2012;28:167-69.

9. Balkan C, Kavakli K, Karapinar D. Spinal epidural haematoma in a patient with haemophilia B: case report. *Haemophilia* 2006;12:437-40.

10. Bisson EF, Dumont T, Tranmer B. Spontaneous spinal epidural hematoma in a child with hemophilia B. *Can J Neurol Sci* 2007;34:488-90.

11. Blount J, Doughty K, Tubbs RS, et al. In utero spontaneous cervical thoracic epidural hematoma imitating spinal cord birth injury. *Pediatr Neurosurg* 2004;40:23-27.

12. Borkar SA, Prasad GL, Dutta Satyarthee G, et al. Spontaneous spinal extradural hematoma in a child with haemophilia B, surgery or medical management – A dilemma? *J Pediatr Neurosci* 2011;6:131-33.

13. Cabral AJ, Barros A, Aveiro C, et al. Spontaneous spinal epidural haematoma due to arteriovenous malformation in a child. *BMJ Case Reports*. 2011:1-4.

14. Cakir E, Karaarslan G, Usul H, et al. Clinical course of spontaneous spinal epidural haematoma mimicking Guillain-Barré syndrome in a child: a case report and literature review. *Devel Med Child Neurol* 2004;46:838-42.

15. Caldarelli M, Di Rocco C, La Marca F. Spontaneous spinal epidural hematoma in toddlers : Description of two cases and review of the literature. *Surg Neurol* 1994;41:325-29.

16. Carrea R, Girado M, Eurnekian A. Hematoma cronico epidural y subdural espinal, relato de tres casos y analisis de la literatura sobre el tema. *Medicina* 1954;14:379-97.

17. Chang CW, Lin LH, Liao HT, et al. Spontaneous spinal epidural hematoma in a 5-year-old girl: case reports. *Acta Paediatr Taiwan* 2002;43:345-47.

18. Chrétiennot-Bara C, Guët A, Balzamo E, et al. Hématome épidural chez l'enfant hémophile: difficultés diagnostiques. *Arch de Pédiatr* 2001;8:828-33.

19. Cirek B, Guven MB, Akalan N. Spontaneous spinal epidural hematoma. *American Congress of Rehabilitation Medicine and the American Academy of Physical Medicine and Rehabilitation* 1999;80:125.

20. Cooper DW. Spontaneous spinal epidural hematoma: Case report. *Journal Neurosurg* 1966;26:343-45.

21. Cromwell LD, Kerber C, Ferry PC. Spinal cord compression and hematoma: An unusual complication in a hemophiliac infant. *Am J Roentgenol* 1977;128:847-49.

22. Cuvelier GDE, Davis JH, Purves EC, et al. Torticollis as a sign of cervico-thoracic epidural haematoma in an infant with severe haemophilia A. *Haemophilia* 2006;12:683-86.

23. Dauch WA. Spinale Epidural-hämatome bei Kindern und Jugendlichen. *Neurochirurgia* 1986;29:83-89.

24. Dawson BH. Paraplegia due to spinal epidural haematoma. *J Neurol Neurosurg Psychiatr* 1963;26:171-73.

25. Doymaz S, Schneider J. Spontaneous spinal epidural hematoma in a teenage boy with cholestasis: A case report. *Pediatr Emerg Care* 2013;29:227-29.

26. Erkutlu I, Bay A, Aktekin E. Conservative management of an acute spinal epidural hemorrhage in a child with hemophilia a with inhibitor. *Blood Coagul Fibrinolysis* 2016;27:930-32.

27. Fok WM, Sun LK, Wong NM, et al. Spontaneous spinal epidural haematoma in a 15-month-old boy presenting with a wry neck: a case report. *J Orthopaed Surg* 2007;15:373-75.

28. Fruchtman Y, Dardik R, Barg AA, et al. Spinal epidural hematoma following cupping glass treatment in an infant with hemophilia A. *Pediatr Blood Cancer* 2016;63:1120-22.

29. Gelabert M, Iglesias M, Gonzalez J, et al. Hematoma epidural espinal espontaneo: revision de 8 casos. *Neurologia* 2003;18:357-63.

30. Ghanem Q, Ivan LP. Spontaneous spinal epidural hematoma in an 8-year-old boy. *Neurology* 1978;28:829-32.

31. Grollmus J, Hoff J. Spontaneous spinal epidural haemorrhage: good results after early treatment. *J Neurol Neurosurg Psychiatr* 1975;38:89-90.

32. Gupta V, Kundra S, Chaudhary AK, et al. Cervical epidural hematoma in a child. *J Neurosci Rural Practice* 2012;3:217-18.

33. Güzel A, Simsek O, Karasalihoglu S, et al. Spontaneous spinal epidural hematoma after seizure: A case report. *Clin Pediatr* 2007;46:263-65.

34. Hamre MR, Haller JS. Intraspinal hematomas in hemophilia. *Am J Pediatr Hematolog/Oncol* 1992;14:166-69.

35. Hasni Bouraoui I, Dhahbi MK, Arifa N, et al. L'hématome spontané épidural rachidien : à propos de 3 cas. *J Radiol* 2009;90:499-502.

36. Heer JS, Enriquez EG, Carter AJ. Spinal epidural hematoma as first presentation of hemophilia A. *Journal Emerg Med* 2008;34:159-62.

37. Hehman K, Norrell H. Massive chronic spinal epidural hematoma in a child. *Am J Dis Child* 1968;116:308-10.

38. Horcajadas A, Katati M, Arraez MA, et al. Hematoma epidural espinal espontaneo: presentacion de 2 casos y revision de la bibliografia. *Neurologia* 1998;13:410-404.

39. Hosoki K, Kumamoto T, Kasai Y, et al. Infantile spontaneous spinal epidural hematoma. *Pediatr Internat* 2010;52:507-8.

40. Hutt PJ, Herold ED, Koenig BM, et al. Spinal extradural hematoma in an infant with hemophilia A: An unusual presentation of a rare complication. *J Pediatr* 1996;128:704-6.

41. Iguchi T, Ito Y, Asai M, et al. A case of spontaneous spinal epidural hematoma. *No to Hattatsu* 1993;25:267-70.

42. Iizuka Y, Iizuka H, Nagano K, et al. Laminoplasty preserving the deep extensor musculature for a pediatric cervicothoracic epidural hematoma case of haemophilia A. *J Pediatr Orthoped* 2014;23:584-88.

43. Irwin SL, Attia MW. Atraumatic spinal epidural hematoma in an infant with hemophilia A. *Pediatr Emerg Care* 2001;17:40-41.

44. Iwamuro H, Morita A, Kawaguchi H, et al. Resolution of spinal epidural haematoma without surgery in a haemophilic infant. *Acta Neurochirurg* 2004;146:1263-65.

45. Jackson R. Case of spinal apoplexy. *Lancet*. 1869;3:5-6.

46. Jackson FE. Spontaneous spinal epidural hematoma coincident with whooping cough. Case report. *J Neurosurg* 1963;20:715-17.

47. Johnston LM. Epidural hemangioma with compression of spinal cord. *JAMA* 1938;11:119-21.

48. Jones RK, Knighton RS. Surgery in Hemophiliacs with Special Reference to the Central Nervous System. *Annals of Surgery* 1956;December:1029-1034.

49. Joseph AP, Vinen JD. Acute spinal epidural hematoma. *J Emerg Med* 1993;11:437-41.

50. Jost W, Graf N, Pindur G, et al. Intraspinale, extradurale Blutung bei einem 7 jährigen Jungen mit Hämophilie B. *Monatsschrift Kinderheilkunde* 1990;138:36-37.

51. Jumani DB, Littlewood R, Iyer A, et al. Spontaneous spinal epidural haematoma mimicking meningitis in a 2-year-old child-a case report and literature review. *Child Nerv Syst* 2013;29:1795-98.

52. Kalina P, Morris J, Raffel C. Spinal epidural hematoma in an infant as the initial presentation of severe hemophilia. *Emerg Radiol* 2008;15:281-84.

53. Khursheed N, Makhdoomi R, Geelani S, et al. Spinal epidural hematoma in a hemophilic child: Case report and literature review. *Neurosurg Quart* 2013;23:222-23.

54. Khursheed N, Makhdoomi R, Tanki H, et al. Spinal epidural hematomas in fast bowlers: Report of two unusual cases. *Pediatr Neurosurg* 2013;49:303-6.

55. Kiehna EN, Waldron PE, Jane Jr JA. Conservative management of an acute spontaneous holocord epidural hemorrhage in a hemophiliac infant: case report. *J Neurosurg Pediatr* 2010;6:43-48.

56. Kirwan R, Saigal G, Faingold R, et al. Nontraumatic acute and subacute enhancing spinal epidural hematoma mimicking a tumor in a child. *Pediatr Radiol* 2004;34:499-502.

57. Kitagawa RS, Mawad ME, Whitehead WE, et al. Paraspinal arteriovenous malformations in children. *J Neurosurg Pediatr* 2009;3:425-28.

58. Kotilainen EM, Pajulo O. Spontaneous epidural hematoma as a cause of sciatic pain in a schoolboy. *Pediatr Neurol* 1997;17:350-52.

59. Kubota T, Miyajima Y. Spinal extradural haematoma due to haemophilia A. *Arch Dis Child* 2007;92:498.

60. Lannum S, Stratton J. Spontaneous epidural hematoma of the thoracic spine in a 17-year-old adolescent boy: a case report. *Am J Emerg Med* 2009;27:628.e5-e6.

61. Lee JS, Yu CY, Huang KC, et al. Spontaneous spinal epidural hematoma in a 4-month-old infant. *Spinal Cord* 2007;45:586-90.

62. Lee CH, Song GS, Kim YH, et al. Spontaneous spinal epidural hematoma coexisting Guillan-Barré syndrome in a child: A case report. *Korean J Spine* 2016;13:167-69.

63. Lehman AAR, McKenna ME, Wisneski R, et al. Delayed presentation of a traumatic spinal epidural hematoma in a preadolescent: A case report. *J Bone Joint Surg* 2011;93:1-4.

64. Lepintre J, Despres P, Rendu, Seringe P. Hématome intra-rachidien extra-dural de siège cervical. *Annales de pédiatrie* 1969;16:723-27.

65. Lepoire J, Tridon P, Montaut J, et al. L'hématome extradural rachidien spontané. *Neurochirurgie* 1959;7:298-313.

66. Liao CC, Hsieh PC, Lin TK, et al. Surgical treatment of spontaneous spinal epidural hematoma: a 5-year experience. *J Neurosurg Spine* 2009;11:480-86.

67. Liao CC, Lee ST, Hsu WC, et al. Experience in the surgical management of spontaneous spinal epidural hematoma. *J Neurosurg Spine* 2004;100:38-45.

68. Licata C, Zoppetti MC, Perini SS, et al. Spontaneous spinal haematomas. *Acta Neurochirurg* 1988;95:126-30.

69. Lim JJ, Yoon SH, Cho KH, et al. Spontaneous spinal Epidural hematoma in an infant : A case report and review of the literature. *J Korean Neurosurg* 2008;44:84-87.

70. Lo MD. Spinal cord injury from spontaneous epidural hematoma. Report of 2 cases. *Pediatr Emerg Care* 2010;26:445-47.

71. Matsumae M, Shimoda M, Shibuya N, et al. Spontaneous cervical epidural hematoma. *Surg Neurol* 1987;28:381-84.

72. Mayer JA. Extradural spinal hemorrhage. Case report. *Canadian Med Assoc J* 1963;89:1034-37.

73. Min S, Duan Y, Zhang L. Chronic spontaneous cervicothoracic epidural hematoma in a 8-month-old infant. *Ann Saudi Med* 2011;31:301-4.

74. Miyagi Y, Miyazono M, Kamikaseda K. Spinal epidural vascular malformation presenting in association with a spontaneously resolved acute epidural hematoma. Case report. *J Neurosurg* 1998;88:909-11.

75. Morsing IE, Brons P, Draaisma JMT, et al. Hemophilia A and spinal epidural hematoma in children. *Neuropediatrics* 2009;40:245-48.

76. Muhonen MG, Piper JG, Moore SA, et al. Cervical epidural hematoma secondary to an extradural vascular malformation in an infant: Case report. *Neurosurgery* 1995;36:585-88.

77. Nagel MA, Taff IP, Cantos EL, et al. Spontaneous spinal epidural hematoma in a 7-year-old girl : Diagnostic value of magnetic resonance imaging. *Clin Neurol Neurosurg* 1989;91:157-60.

78. Narawong D, Gibbons VP, McLaughlin JR, et al. Conservative management of spinal epidural hematoma in haemophilia, Case reports. *Pediatr Neurol* 1988;4:169-71.

79. Nayak N, Baldawa S, Diyora B, et al. Delayed recovery of paraplegia following surgical evacuation of spontaneous cervicothoracic epidural hematoma. *Childs Nerv Syst* 2011;27:2031-32.

80. Nichols P, Manganiello LOJ. Extradural hematoma of the spinal canal. Report of a case. *Case Reports* 1956:638-40.

81. Nirupam N, Pemde H, Chandra J. Spinal epidural hematoma in a patient with hemophilia B presenting as acute abdomen. *Ind J Hematol Blood Transf* 2014;30:54-56.

82. Noth I, Hutter J, Meltzer PA, et al. Spinal epidural hematoma in a hemophilic infant. *Am J Pediatr Hematol/Oncol* 1993;15:131-34.

83. Ohaegbulam CO, Dunn IF, d'Hemecourt P, et al. Lumbar epidural hematoma associated with spondylolyses. Report of 3 cases. *J Neurosurg Spine* 2008;8:174-80.

84. Oymak Y, Muminoglu N, Ay Y, et al. Torticollis in a haemophilic infant with inhibitor: a case of spinal epidural haematoma. *Blood Coagulation Fibrinolysis* 2016;27:576-79.

85. Packer NP, Cummins BH. Spontaneous epidural haemorrhage: a surgical emergency. *Lancet* 18:356-58.

86. Pai SB, Maiya PP. Spontaneous spinal epidural hematoma in a toddler-a case report. *Childs Nerv Syst* 2006;22:526-29.

87. Paraskevopoulos D, Magras I, Polyzoidis K. Spontaneous spinal epidural hematoma secondary to extradural arteriovenous malformation in a child: a case-based update. *Childs Nerv Syst* 2013;29:1985-91.

88. Patel H, Garg BP. Increasing irritability with sudden onset of flaccid weakness. *Seminars Pediatr Neurol* 1996;3:192-97.

89. Patel H, Boaz JC, Phillips JP, et al. Spontaneous spinal epidural hematoma in children. *Pediatr Neurol* 1998;19:302-7.

90. Pear BL. Spinal epidural hematoma. *Radiol Conf Rocky Mountain Radiol Soc* 1972;115:155-65.

91. Pecha MD, Able AC, Barber DB, et al. Outcome after spontaneous spinal epidural hematoma in children : Case report and review of the literature. *Arch Physical Med Rehab*1998;79:460-63.

92. Per H, Canpolat M, Tümtürk A, et al. Different etiologies of acquired torticollis in childhood. *Childs Nerv Syst* 2013;13:2302-06.

93. Poonai N, Rieder MJ, Ranger A. Spontaneous spinal epidural hematoma in an 11-month-old girl. *Pediatr Neurosurg* 2007;43:121-24.

94. Posnikoff J. Spontaneous spinal epidural hematoma of childhood. *J Pediatr* 1968;73:178-83.

95. Rajz G, Cohen JE, Harnof S, et al. Spontaneous spinal epidural hematoma: The importance of preoperative neurological status and rapid intervention. *J Clin Neurosci* 2015;22:123-28.

96. Ramelli GP, Boscherini D, Kehrli P, et al. Spontaneous spinal epidural hematomas in children : Can we prevent a negative prognosis ? Reflections on 2 cases. *J Child Neurol* 2008;23.

97. Rao BD, Rao KS, Subrahmanian MV, et al. Spinal epidural haemorrhage. *Br J Surg* 1966;53:649-50.

98. Rathi N, Rathi A. Haemophilia A presenting as spontaneous spinal epidural haematoma in a 3-month-old infant. *BMJ Case Reports* 2010;10:1-3.

99. Ratre S, Yadav Y, Choudhary S, et al. Spontaneous ventral spinal epidural hematoma in a child: A case report and review of literature. *J Neurosci Rural Practice* 2016;7:297-99.

100. Ravid S, Schneider S, Maytal J. Spontaneous spinal epidural hematoma: an uncommon presentation of a rare disease. *Childs Nerv Syst* 2002;18:345-47.

101. Rebello MD, Dastur HM. Spinal epidural haemorrhage. A review and two case reports. *Neurol India* 1966;14:135-45.

102. Robertson WC, Lee YE, Edmonson MB. Spontaneous spinal epidural hematoma in the young. *Neurology* 1979;29:120-22.

103. Rois PV, Lopez MR, Lopez de Vergara BC, et al. Spinal epidural hematoma in hemophilic children: controversies in management. *Childs Nerv Syst* 2009;25:987-91.

104. Rosenberg O, Itshayek E, Israel Z. Spontaneous spinal epidural hematoma in a 14-year-old girl: case report and review of the literature. *Pediatr Neurosurg* 2003;38:216-18.

105. Sandvik U, Svensdotter E, Gustavsson B. Spinal cavernous extradural angiolipoma manifesting as spontaneous spinal epidural hematoma in a child. *Childs Nerv Syst* 2015;31:1223-26.

106. Sankar J, Srinivasan A, Ramakrishnan V, et al. An unusual cause of acute torticollis. *Clin Pediatr* 2009;48:784-95.

107. Scheil F, Larkamp U, Mutharoglu U. Das spontane spinale epidurale Hämatom. *Neurochirurgia* 1990;33:45-49.

108. Schoonjans AN, De Dooy J, Kenis S, et al. Spontaneous spinal epidural hematoma in infancy : Review of the literature and the seventh case report. *Eur J Paediatr Neurol* 2013;17:537-42.

109. Scott TE. Spinal epidural hemorrhage: Spontaneous and recurrent. *Southern Med J* 1958;51:1048-50.

110. Sheikh AA, Abildgaard CF. Medical management of extensive spinal epidural hematoma in a child with factor IX deficiency. *Pediatr Emerg Care* 1994;10:26-29.

111. Shenkin HA, Horn RC, Grant FC. Lesions of the spinal epidural space producing cord compression. *Arch Surg* 1945;51:140-42.

112. Shin J-J, Kuh S-U, Cho Y-E. Surgical management of spontaneous spinal epidural hematoma. *Eur Spine J* 2006;15:998-1004.

113. Shin Y-I, Yang C-Y, Joo M-C, et al. Spontaneous epidural hematoma recurred three times. *Neurorehabilitation* 2007;22:147-50.

114. Sivakumaran R, King A, Bodi I, et al. Spontaneous epidural spinal haematoma in children caused by vascular malformations. *Eur Spine J* 2016;25:614-18.

115. Soundappan SVS, Darwish B, Chaseling R. Traumatic spinal epidural hematoma – Unusual cause of torticollis in a child. *Pediatr Emerg Care* 2005;21:847-49.

116. Stanley P, McComb JG. Chronic spinal epidural hematoma in hemophilia A in a child. *Pediatr Radiol* 1983;13:241-43.

117. Tagaya A, Miyajima Y, Sakamoto M, et al. Spontaneous spinal epidural hematoma in an infant with undiagnosed hemophilia A. *Pediatr Internat* 2010;52:296-318.

118. Tamura K, Kanazawa T, Suzuki M, et al. Successful induction of immune tolerance by continuous infusion of recombinant factor VIII in a haemophilia A patient with high-inhibitor titres. *Haemophilia* 2006;12:100-2.

119. Tender GC, Awasthi D. Spontaneous cervical spinal epidural hematoma in a 12-year-old girl : Case report and review of the literature. *J Louisiana State Med Soc* 2004;156:196-98.

120. Tewari MK, Tripathi LN, Mathuriya SN, et al. Spontaneous spinal extradural hematoma in children: Report of three cases and review of the literature. *Childs Nerv Syst* 1992;8:53-55.

121. Travis SF, Greenbaum BH, Mintz M, et al. Epidural hematoma of the cauda equina in a child with hemophilia A. *J Pediatr Hematol/ Oncol* 1996;18:308-10.

122. Valladares HA. Spinal epidural hematoma. Report of 2 cases. *Neurochirugia* 1972;30:236-43.

123. Vallee B, Besson G, Gaudin J, et al. Spontaneous spinal epidural hematoma in a 22-month-old girl. Case report. *J Neurosurg* 1982;56:135-38.

124. Van Heesewijk JPM, Casparie JWBM. Acute spontaneous spinal epidural haematoma in a child : case report. *Europ Radiol* 2000;10:1874-76.

125. Ventureyra ECG, Ghanem Q, Ivan LP. Spontaneous spinal epidural haematoma in a youngster. *Child's Brain* 1979;5:103-8.

126. Visconti EB, Hilgartner MW. Recognition and management of central nervous system hemorrhage in hemophilia. *Paediatrician* 1980;9:127-37.

127. Williams CE, Nelson M. The varied computed tomographic appearances of acute spinal epidural haematoma. *Clin Radiol* 1987;38:363-65.

128. Wittebol MC, Van Veelen CWM. Spontaneous spinal epidural haematoma : Etiological considerations. *Clin Neurol Neurosurg* 1984;86:265-70.

129. Yildiz S, Dokumaci DS, Boyaci N, et al. Spontaneous thoracic epidural hematoma mimicking spinal mass. *The spine journal* 2014;14.

130. Zhong W, Chen H, You C, et al. Spinal epidural hematoma in a child with hemophilia B. *Neurol India* 2011;59:294-96.
